# Temperance and Education

**Published:** 1902-09-27

**Authors:** 


### Temperance and Education.

It is one of the gravest misfortunes of the modern government of many countries that those who are charged with conducting it live mainly under bondage to fools. In other words, they are compelled to subordinate their knowledge to the ignorance of the numerical majority, as the sole condition under which they will be permitted to continue governing; and they must be perfectly well aware, in the greater number of instances, that the ignorance of the majority is seriously prejudicial to the welfare of the State. To proud men, or even to highly conscientious men, the position thus created would be intolerable; but the pursuit of practical politics has no tendency to encourage pride, and is perhaps not eminently favourable to the excessive growth of conscience. However this may be, professional politicians are as a rule content to follow the trade union principle of "going easy"; and, if they cannot carry what they know to be right, are perfectly willing to accept what they know to be wrong. They intend, let us say, to carry an Education Bill, and, if this should be divested by successive amendments of nearly all the useful provisions of the original draft, they will still consider that by placing an Act of some sort upon the Statute Book they have fulfilled their pledges to the country. At the present time there is a remarkable consensus of opinion, among those who know, to the effect that the position of the United Kingdom is seriously threatened by the competition of nations better educated than our own; that is to say, in whom the great bulk of the rising generation have been rendered better able than the corresponding classes among ourselves to comprehend and to take advantage of the daily occurring increase of scientific knowledge, and to apply that knowledge to the technicalities of many arts, in such a manner as to cheapen processes and to improve results. There is no question of the production or development of original genius, but merely of the definite elevation of the standard of knowledge of the average individual. The condition at which we have arrived is due to many causes, but chiefly, perhaps, to the abysmal ignorance of the common University graduate, who turns schoolmaster, with regard not only to the value of science in general, but more especially to its bearing upon the duties of his own craft. Dr. Dukes once wrote something to the effect that the conduct of schools for the upper and middle classes of this country is the only calling in life which is not thought to require any special knowledge or preparation; and it may fairly be assumed that the ambition of the average master of a Board School is to follow in the footsteps of his professional brother of higher social pretensions. The general result is that which all who are familiar with it are unanimous in deploring.

While this is the general state of things, and while our various theological Mrs. Partingtons are bringing their best mops for the arrest of the great incoming tide of knowledge, attention has been called in a curious way to one little side issue which is of special interest to the medical profession. A gentleman named Morris, who is in some capacity an official representative of antagonism to the use of alcoholic drink, has been writing to the *Times* to describe the very elaborate arrangements in many of the schools of the United States for teaching "temperance" to children, and he was able to bring forward a considerable list of schools and localities in which such teaching forms a conspicuous part of the authorised programme. He has been met by another correspondent, whose identity is veiled under the modest *nom de plume* of "X," and who pertinently calls attention to the observations on this subject made by Miss Alice Ravenhill, in her report to the Sanitary Institute on the schools of the United States, a report which we have already mentioned as containing much valuable information. Miss Ravenhill calls attention to the fact that a body described as the "Women's Temperance Union" has been able to exert such pressure upon various State Legislatures as not only to obtain an enactment that one-third of the time devoted in the school course to "elementary physiology" shall be applied to teaching the injurious effects of alcohol and of narcotic drugs upon the human system, but also to secure that the text-books employed in teaching physiology shall be submitted to this "Union" for approval, and for rejection if their teaching on the alcohol question is not satisfactory to a body which Miss Ravenhill mildly describes as being composed of "ardent partisans." One consequence of this arrangement seems to be an impossibility of procuring good physiological text-books, the authors capable of producing them being properly unwilling to write to the order of a non-physiological tribunal; while another consequence is that the books which are authorised so disgust the more rational teachers and superintendents as to induce them to avoid the subject of physiology altogether, or at least to deal with it in a merely perfunctory manner, and in compliance with the letter of the rules under which they work. One can almost imagine the pupils hastening to get drunk, if only for the purpose of ascertaining by experiment whether the magnitude of the evils incidental to the use of alcohol had not been exaggerated by their teachers.

